# How well can the exponential-growth coalescent approximate constant-rate birth–death population dynamics?

**DOI:** 10.1098/rspb.2015.0420

**Published:** 2015-05-07

**Authors:** Tanja Stadler, Timothy G. Vaughan, Alex Gavryushkin, Stephane Guindon, Denise Kühnert, Gabriel E. Leventhal, Alexei J. Drummond

**Affiliations:** 1 Department of Biosystems Science and Engineering, ETH Zürich, Zürich, Switzerland; 2 Institute of Integrative Biology, ETH Zürich, Zürich, Switzerland; 3 Swiss Institute of Bioinformatics (SIB), Lausanne, Switzerland; 4 Department of Computer Science, The University of Auckland, Auckland, New Zealand; 5 Department of Statistics, The University of Auckland, Auckland, New Zealand; 6 Allan Wilson Centre for Molecular Ecology and Evolution, Palmerston North, New Zealand; 7 Institute of Veterinary Animal and Biomedical Sciences, Massey University, Palmerston North, New Zealand; 8 LIRMM, UMR 5506, Montepellier, France

**Keywords:** phylodynamics, phylogenetics, epidemiology, population genetics, birth–death model

## Abstract

One of the central objectives in the field of phylodynamics is the quantification of population dynamic processes using genetic sequence data or in some cases phenotypic data. Phylodynamics has been successfully applied to many different processes, such as the spread of infectious diseases, within-host evolution of a pathogen, macroevolution and even language evolution. Phylodynamic analysis requires a probability distribution on phylogenetic trees spanned by the genetic data. Because such a probability distribution is not available for many common stochastic population dynamic processes, coalescent-based approximations assuming deterministic population size changes are widely employed. Key to many population dynamic models, in particular epidemiological models, is a period of exponential population growth during the initial phase. Here, we show that the coalescent does not well approximate stochastic exponential population growth, which is typically modelled by a birth–death process. We demonstrate that introducing demographic stochasticity into the population size function of the coalescent improves the approximation for values of *R*_0_ close to 1, but substantial differences remain for large *R*_0_. In addition, the computational advantage of using an approximation over exact models vanishes when introducing such demographic stochasticity. These results highlight that we need to increase efforts to develop phylodynamic tools that correctly account for the stochasticity of population dynamic models for inference.

## 1. Introduction

The composition of individuals that make up a population often changes through time. In many cases, mathematical models can be formulated that describe the qualitative dynamics of the population. However, in order to obtain a more quantitative description, statistical methods are required that can estimate model parameters from population data. Genetic sequence data, in particular, have been invaluable at informing the dynamical processes that shape populations. Such processes include for example environmental fluctuations [1], speciation events (see for example recent reviews by Pyron & Burbrink [2] and Stadler [3]) and in particular the infection of new hosts in the case of infectious diseases (reviewed by Kühnert *et al.* [4]).

At the core, such methods use the fact that genetic sequences obtained from individuals within the population differ enough to reconstruct their genealogy. In a genealogy, a coalescent event represents the most recent ancestor of two (or more) lineages. Now, typically the genealogy is assumed to be equal to the phylogeny of the population history. In a phylogeny, a branching event represents a birth event in the population. By equalling the genealogy to the phylogeny, we assume that a coalescent event in the genealogy corresponds to a birth event in the population. The reconstructed phylogeny (genealogy) now is used to quantify the parameters of the population dynamic process (birth and death of individuals in the population). Surprisingly, genetic information from only few individuals can lead to deep insights into the population dynamics as a whole (for an overview, see [4]).

The time resolution of this phylogeny depends on the time scale at which genetic mutations occur versus the time scale at which the population composition changes. When both these changes co-occur (i.e. when the evolutionary time scale is comparable to the time scale of the population dynamics), then there is a close correspondence between the population genetic and population dynamic history [5]. In the case of an infectious pathogen, the reconstructed phylogeny can be interpreted as a proxy for an incomplete transmission tree. In such an incomplete transmission tree, branches that link two individuals represent chains of transmission from one individual to another, which may or may not have involved unobserved intermediate individuals. The incomplete transmission tree can then be used to inform models of the population dynamics [6–12].

Phylogenetic reconstruction methods originally made simplifying assumptions that are common in the field of population genetics. The Wright–Fisher and Moran models, for example, assume that the number of individuals remains fixed through time. Using the coalescent framework, it is then possible to derive the likelihood for any specific phylogenetic tree that can be used in likelihood-based inference methods [13]. It is important to note that while the coalescent is typically used to model genealogies, here the coalescent is a model for the phylogenetic tree (i.e. the population dynamics). The accuracy of a reconstructed phylogeny obtained using this framework (and by proxy a—typically incomplete—transmission tree in the case of infectious disease outbreaks), however, will strongly depend on the validity of the assumptions underlying the coalescent approximation. A major advance in coalescent theory was introduced by Griffiths & Tavaré [14], who generalized the coalescent to population sizes that can be described by an integrable function through time. Central to the derivation of both the original constant population size formulation of Kingman [13] and the parametric coalescent of Griffiths & Tavaré [14] is the assumption that the number of sampled individuals remains fixed and the population size is large compared with the sample size, irrespective of whether the discrete generation Wright–Fisher or Moran model is used [15]. Fu [16] showed that even though the Kingman coalescent derivation is obtained for small sample sizes, the Kingman coalescent is also a good approximation to the population dynamics for bigger sample sizes. Boskova *et al.* [17] hypothesize that the key assumption of the coalescent often being violated is stochastically changing population size through time.

The joint inference of the phylogeny as well as the model for the population dynamics is called ‘phylodynamic inference’ and has become popular in a variety of fields, including the study of infectious disease outbreaks [7,8,18–20]. Phylodynamic inferences mostly rely on the coalescent to approximate the underlying population dynamics. In particular, implementations assuming both parametric [7,8,18,21] and non-parametric [22,23] population size changes are commonly used for statistical inference under the coalescent, and the inferred population size changes shed light on the population dynamic process. However, the justification of the coalescent assumptions is often questionable in the case of epidemic outbreaks. Specifically, epidemic outbreaks commonly originate from a single individual. Thus, in the early stages of the outbreak, the number of infected individuals (i.e. the population size in the coalescent) is small, and therefore cannot be much larger than the number of sampled individuals.

Key to most epidemiological models of infectious dynamics (e.g. SIS, SIR, SEIR) is a phase of (exponential) population growth from the initial infected individual. These compartmental models can be written as forward-in-time birth–death (BD) models, with the initial exponential growth phase being a constant-rate BD model. Thus, deterministic exponential-growth coalescent models [24] appear as an appropriate description of early outbreaks. The deterministic exponential-growth coalescent has been used to estimate the initial growth rate, and from that the basic reproductive number *R*_0_ of the pathogen [7].

Recent work has proposed a direct modelling of stochastic epidemiological dynamics (e.g. SIS or SIR dynamics) for phylogenetic inference that avoids any approximations made in the coalescent framework [4,9,11,12]. These models are forward-in-time stochastic BD models explicitly modelling transmission, recovery and sampling. These approaches build upon the BD framework that is commonly used to reconstruct evolutionary relationships between species [25–29]. The advantage of these methods is that they do not implicitly require the assumptions of the coalescent to be justified, but assume stochastic epidemiological models such as SIS or SIR; however, they are often computationally much more expensive than many of the coalescent-based approximations.

Our aim here is to investigate the applicability of the coalescent approximation when performing phylodynamic inference. We assume that epidemic outbreaks are described by a constant-rate BD model, which is a good approximation to the early phases of most well-established epidemiological models [30,31]. We show that the coalescent approximation assuming an exponentially varying population size fails to accurately retrieve the true distribution of coalescent times of two randomly chosen individuals. We also show that replacing the deterministic exponential growth function with an ensemble of trajectories that are sampled from the full stochastic BD model in the coalescent approximation yields a good approximation to the true distribution. The added computational cost of such a stochastic extension of the coalescent, however, sacrifices its primary advantage compared with birth–death approaches. In fact, direct application of BD models is much more computationally efficient than the coalescent approximation for simple stochastic exponential growth.

While written in epidemiological terms, our results hold for any population dynamics, which is assumed to grow exponentially under a constant-rate BD model (e.g. species radiation or a rapid spread of languages).

## 2. Models

Our reference model of population growth is the constant-rate BD model [32]. For disease outbreaks, the BD model is the simplest stochastic model of infectious disease dynamics in large susceptible populations with an infectious force proportional to the number of infected individuals. We highlight here that deterministic models for epidemic spread cannot be employed as they assume continuous population sizes, while we have discrete changes in number of infected individuals at branching/removal events. Our model, the constant-rate BD model, assumes that infected individuals have a constant rate of transmitting to susceptibles, *λ*, and a constant rate of becoming uninfected, *μ*. The expected population size or number of infected individuals in this model grows exponentially, which is characteristic of the early phases of most epidemic outbreaks.

We compare the BD model with the coalescent model. In what follows, we refer to a ‘coalescent model’ when talking about the stochastic process giving rise to trees with a coalescent probability distribution (while typically the coalescent is termed a statistical framework rather than a model). It is important to distinguish two views of time here. Time runs in the usual (forward) sense when we consider population growth models, such that the value of a time *t*_future_ in the future is larger than the value of time *t*_0_ at the present, *t*_future_ > *t*_0_. When we consider ancestral processes that reconstruct the phylogeny of the population, time runs in the opposite (backward) direction, such that a value of *τ*_past_ in the past is larger than a value of *τ*_0_ at the present, *τ*_past_ > *τ*_0_ (in order to avoid confusion, we denote forward time by the variable *t* and backward time by the variable *τ*).

We compare the ‘time of coalescence’ of two present-time lineages under the BD model with two coalescent-type models with exponentially growing populations. Note that the time of coalescence of two lineages is the most recent time in the past at which these two lineages shared a common ancestor. The lineages may have evolved under any model, not only a coalescent model (despite the name ‘time of coalescent’).

**Figure 1.**
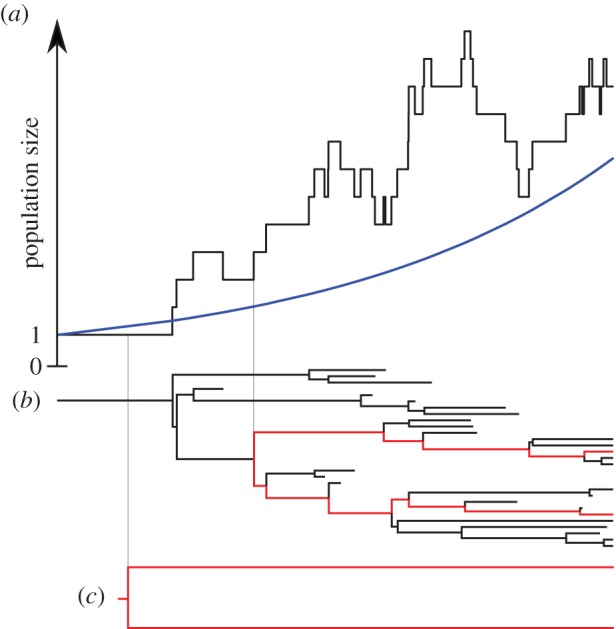
(*a*) Birth–death population size trajectory (black line) and corresponding deterministic exponential growth (blue line) curve obtained with growth rate *r* = *λ* − *μ*. (*b*) Full corresponding birth–death tree (black) and a subtree (red) spanning two sampled lineages. (*c*) A representative and deterministic growth coalescent tree. Note that while coalescence time in the sampled birth–death tree corresponds precisely with a birth event in the population size trajectory, the same is not true for the deterministic coalescent tree.

For the first coalescent model, we consider a standard coalescent model with deterministic exponential population growth [14,24,33]. For the second coalescent model, we define a coalescent model with stochastic population growth [10], where the population trajectories are realizations of a stochastic BD process. Figure 1 shows a realization of a BD population size trajectory compared with the corresponding deterministic exponential population size curve, with exemplary two-leaf subtrees.

Here, we define the BD and the two coalescent models formally, and derive characteristics of the models that facilitate their comparison.

### (a) The BD model

We consider a constant-rate BD model with constant infectious force (birth rate), *λ*, and constant removal (death) rate, *μ* (0 ≤ *μ* < *λ*). The process starts with one initially infected individual and stops after time *T* since the start of the epidemic (*T* is also called time since origin). This is a simple continuous-time epidemiological model that preserves exponential growth of the number of infected individuals while properly accounting for discrete population sizes and allowing for the possibility of early termination of the epidemic. The early phases of SIS- and SIR-type epidemic outbreaks are typically modelled by such a process. Key epidemiological parameters can be derived from the BD model parameters, such as the basic reproductive number [30], *R*_0_ = *λ*/*μ*, and the net growth rate *r* = *λ* − *μ*.

In order to determine the expected population growth and the coalescent time distribution under the BD process, we define *p*_0_(*t*) as the probability that a single individual has no extant offspring after time *t*, and *p*_1_(*t*) as the probability that a single individual has exactly one extant offspring after time *t*. Following Kendall [34],

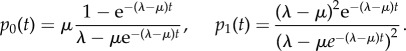


#### (i) Expected population size under the BD model

The expected population size under the BD model grows exponentially,
2.1
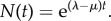


The BD process is an individual-based model, with each individual having some rate of dying. Thus, it can happen that all individuals die, and the trajectory of the BD process does not ‘survive’ until time *T*. When analysing a phylogeny of age *T*, we know that the process survived until time *T*. We thus only consider population size trajectories that survive until time *T* (i.e. that have a non-zero population size until time *T*). The expected population size for 0 ≤ *t* ≤ *T* is given by
2.2
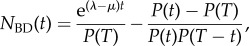
with 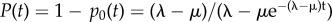 [35]. The conditioning on survival results in an early rapid increase in the expected number of individuals, called ‘push of the past’, after which the population size grows exponentially with rate *r* = *λ* − *μ* [35]. The time *T* such that the expected number of individuals is equal to *N* is
2.3
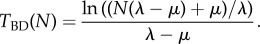


#### (ii) Distribution of coalescent times under the BD model

In the electronic supplementary material, we show that the distribution of time to coalescence (where time is measured backward from the present) of two randomly chosen individuals at time *τ* given parameters *λ*, *μ* and time since origin *T* under the BD model, denoted by *f*_BD_(*τ*), is
2.4
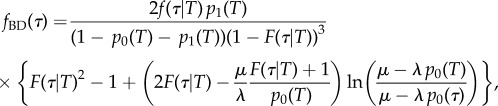
with *f*(τ|*T*) = *μp*_1_(*τ*)/*p*_0_(*T*) and *F*(*τ|T*) = *p*_0_(*τ*)/*p*_0_(*T*). Note that *f*_BD_(τ) ≥ 0 for *τ* ∈ [0, *T*], and *f*_BD_(τ) = 0 for *τ* > *T* since the process started at time *T* in the past.

### (b) The coalescent model with deterministic exponential population growth (CD model)

In the coalescent approximation, two lineages coalesce with rate 1/(*N*(*τ*)*ρ*), with *N*(*τ*) being the population size and *ρ* being the generation time. Thus, the coalescent is defined by parameters *N*(*τ*) and *ρ*, compared with *λ*, *μ*, *T* in the BD process. The coalescent with a deterministic exponentially growing population size (referred to hereafter as the CD model) is therefore defined by the following two parameters: (i) the population growth rate *r* and (ii) a factor *Θ* = *N*_0_*ρ*, where *N*_0_ is the present-day population size *N*_0_, and *ρ* the generation time [14,24]. This yields *N*(*τ*) = *N*_0_e^−*rτ*^.

#### (i) Expected population size growth under the CD model

Because population growth under the CD model is a deterministic process, the (expected) population size at time *t* is just
2.5
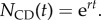


Note that compared with the BD model, there is no extinction of a population.

#### (ii) Distribution of coalescent times under the CD model

The time of coalescence of two lineages picked from a population of size *N* has the probability density [14,24,36]
2.6
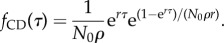


Thus, the probability that the two lineages coalesce between the present and time *τ* in the past is
2.7
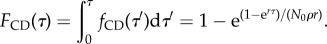


Note that the deterministic coalescent is naturally defined for times *τ* ∈ (0, ∞) before the present, in particular for *τ* > *T* (i.e. prior to the start of the corresponding BD model).

### (c) Link between the BD and CD models

Both the BD and the CD models describe populations that (in expectation) grow exponentially in time. In fact, by comparing equations (2.1) and (2.5), it is natural to set the growth rate in the CD model to *r* = *λ* − *μ*. The expected population size in the BD model at time *t* (unconditioned on survival of the process) is equal to the population size in the CD model at time *t*. Furthermore, *N*_0_ is the present-day population size and relates to the BD parameters via *N*_0_ = e^(*λ*−*μ*)*T*^.

When the coalescent approximates a BD process, the generation time *ρ* must be related to the per capita birth rate *λ*. Volz *et al.* [8] showed that *ρ* = 1/(2*λ*). We explain the derivation of this result in the electronic supplementary material.

#### (i) Breakdown of the CD model

The length of the process *T* required to reach *N*_0_ individuals is then given by *T* = ln*N*_0_/*r*. From the point of view of the ancestral process, at time *T* in the past, the population size was 1. Thus, *T* is the maximum time at which all lineages should have coalesced to one lineage. However, we show in the following that under the CD model, the probability of observing a coalescent event prior to the origin of the process at time *T* in the past is strictly greater than zero.

The rate of coalescence for a changing population size is 1/(*N*(*τ*)*ρ*). The probability that two lineages coalesce to one lineage within time interval [0, *T*] is (equation (2.7))
2.8
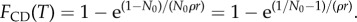


From this expression, we directly observe
2.9
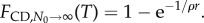


As in our setting *ρ* = 1/2*λ*, we have *ρr* = (*λ* − *μ*)/2*λ* and,

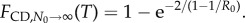


Note that for *μ* = 0 the probability of a coalescent event being ancestral to time *T* is e^−2^ = 0.135, and with increasing *μ* this probability decreases. For *μ* → *λ*, all coalescent events occur between the present and time *T* in the past with probability 1. The case *μ* → *λ* (i.e. *r* → 0) corresponds to constant population size though, so the population size decreases to 1 with probability 0.

### (d) Deterministic coalescent with modified *N*(*t*) (CDN model)

We further investigated the performance of a deterministic coalescent using population size *N*_BD_(*t*) from equation (2.2) instead of *N*_CT_(*t*) from equation (2.5), to make expected population sizes under the BD and coalescent model equivalent. In the electronic supplementary material, we show that the coalescent time probability density under the coalescent with population size function *N*_BD_(*τ*) from equation (2.2) is
2.10
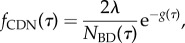


with,



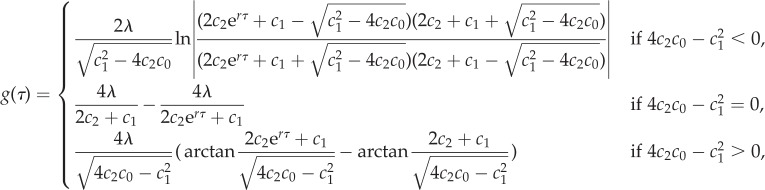


and

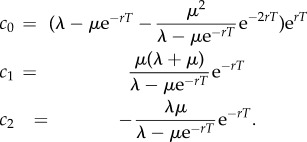


We highlight that not all lineages coalesce by time *T*. As population size is not defined for *τ* > *T*, there is a non-zero probability for no coalescence at all.

### (e) The coalescent with stochastic population growth via birth–death trajectories (CS model)

As pointed out above, the coalescent with a deterministically changing population size does not take into account stochastic fluctuations, which in particular may lead to population extinctions. In order to employ a coalescent with such stochastic population size changes, we define a coalescent process where population size is a population trajectory of the BD model, and the rates of coalescence within this population are 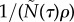 [20], where 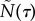 is the population size at time *τ* in the trajectory. We refer to this model as a stochastic coalescent (hereafter CS).

As before, the generation time is *ρ* = 1/(2*λ*). Because the population size is undefined ancestral to time *T*, we assign 0 probability to coalescent times older than *T*. In fact, although *T* is not a parameter in the deterministic coalescent (as lineages simply coalesce backwards in time until the most recent common ancestor of the sample is found), it is a natural parameter for the stochastic coalescent, as BD trajectories are simulated for time *T*. However, note that under the stochastic coalescent model, lineages may not all coalesce when tracing them back in time for a given population size trajectory.

#### (i) Expected population size growth

The expected population size growth follows equation (2.2).

#### (ii) Distribution of coalescent times

While analytical derivation of the distribution of the time to coalescent exists when the population size varies according to a stationary Markov process [37], no such result is available for non-stationary stochastic processes, which is the case here. We therefore obtained the probability density of coalescent times *f*_CS_(*t*) under the stochastic coalescent via simulations. We first simulated BD population size trajectories forward in time for a duration of time *T*. Then, we sampled the coalescent time of two lineages extant at the present backward in time.

## 3. Results

**Figure 2.**
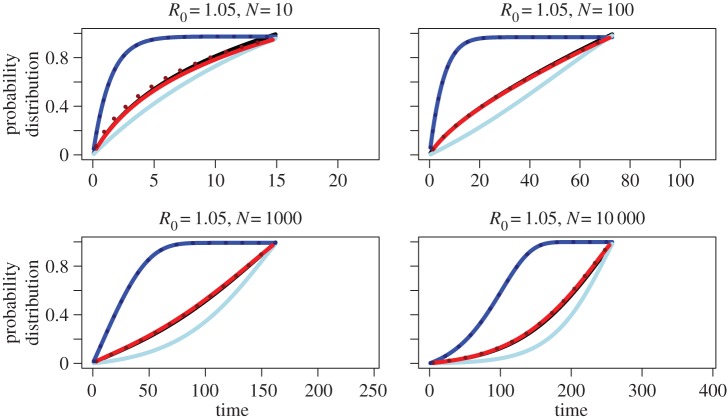
Cumulative probability distribution function of time to coalescence of two lineages in our models, *f*_BD_(*t*), *f*_CD_(*t*), *f*_CDN_(*t*) and *f*_CS_(*t*), for low *R*_0_ = 1.05 and *N* = 10, 100, 1000 and 10 000. Black displays the distribution of coalescent times under the birth–death (BD) model, blue under the deterministic coalescent (CD; dotted line corresponds to coalescent rate proportional to 1/(*N*(*t*) − 1)), and red under the stochastic coalescent (CS; dotted line corresponds to coalescent rate proportional to 1/(*N*(*t*) − 1)). Light blue corresponds to the deterministic coalescent with population size being the expected BD population size (CDN).

**Figure 3.**
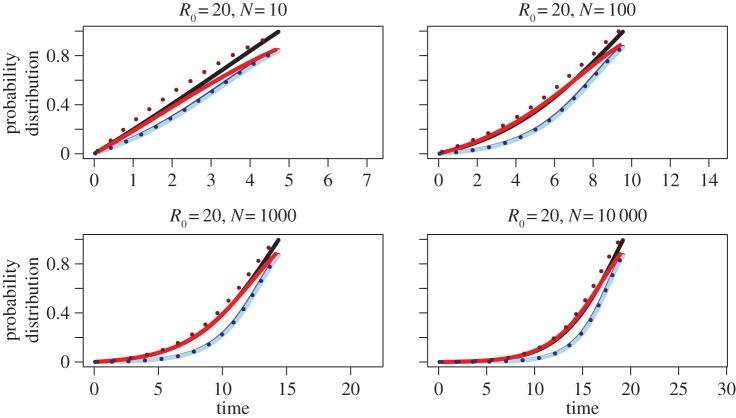
Cumulative probability distribution function of time to coalescence for high *R*_0_ = 20, and *N* = 10, 100, 1000 and 10 000. For details, see the caption of figure 2.

Without loss of generality, we set *λ* = 0.5 (defining our time unit) and thus *ρ* = 1/(2*λ*) = 1, meaning only parameters *μ* and *T* are free to vary. We compare the probability distributions corresponding to the probability densities of the different models, *f*_BD_(*τ*), *f*_CD_(*τ*), *f*_CDN_(*τ*), *f*_CS_(*τ*), for *R*_0_ = 1.05, 1.3, 1.6, 2, 4, 10, 20. Furthermore, we plot in dashed lines the coalescent probability densities when using the coalescent rate proportional to *N* − 1 rather than to *N* (see §2g above). We choose *T* = *T*_BD_(*N*_0_) such that the expected population size at the end of the process is *N*_0_ = 10, 100, 1000, 10 000 (equation (2.3)). The results are summarized in figure 2 for *R*_0_ = 1.05 and figure 3 for *R*_0_ = 20. The plots for the remaining *R*_0_ values are displayed in the electronic supplementary material, figures S1–S5.

In the following, we discuss under which circumstances coalescent time distributions under the coalescent models and the BD models are similar. Under these circumstances, the coalescent is a good approximation to the BD model.

### (a) Deterministic coalescent

Based on plotting coalescent time distributions, we observe that for small values of *R*_0_ (roughly *R*_0_ < 2), coalescent events under the deterministic coalescent (CD; blue line) are younger (occur earlier going backward in time) than under the birth–death (BD; black line) and stochastic coalescent (CS; red line) models (figures 2 and 3). For large values of *R*_0_ (roughly *R*_0_ > 2), the CD model predicts older coalescent events (occurring later going backward in time) than under the BD and CS models.

For large values of *R*_0_, the CD model has a bias towards older coalescent events. This is most likely to be due to the fact that the CD model allows for two lineages to coalesce at a time *τ* > *T* before the start of the process. We can quantify the proportion of coalescent events ancestral to *T* under the deterministic coalescent. The probability that two lineages coalesce before the start of the process (*τ* > *T*) is always larger than zero (equation (2.8)), even when *N*_0_ → *∞*, under which assumption the coalescent approximation was derived (equation (2.9)). When *R*_0_ → ∞, the probability that two lineages coalesce before the start of the process tends to e^−2^ ≈ 0.135. This probability decreases with *R*_0_, and tends to zero for *R*_0_ → 1, meaning that all coalescent events happen in [0, *T*] (though in this case *T* = ∞).

**Figure 4.**
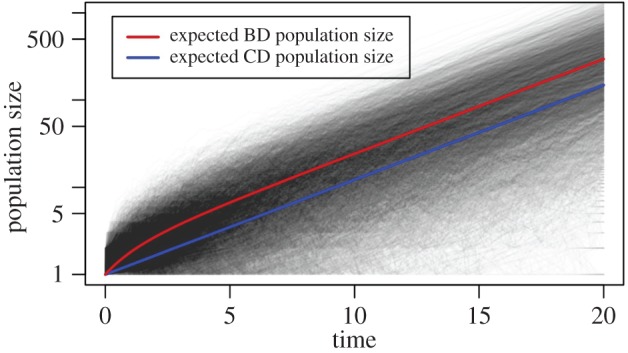
Trajectories of the population size (on a log scale) through time under the birth–death model (black) together with their expected size conditional on survival of the epidemic (red). These trajectories are also the basis of the coalescent with stochastic population growth via birth–death trajectories. The population size under the coalescent with deterministic population size change follows the blue line (which has slope *r* = *λ* − *μ*).

Coalescent times are younger for small *R*_0_ under the deterministic model compared with the stochastic models owing to the differences in population growth. The expected population size at time *t* under the stochastic population size model (equation (2.2)) is larger than under the deterministic model (equation (2.5); see also figure 4). These differences in expected population size are largest for small values of *R*_0_, and tend to zero as *R*_0_ increases to infinity. The rate of coalescence of two lineages is proportional to 1/*N*(*t*), and hence increases with decreasing population size. Therefore, coalescent events in the deterministic model happen faster (smaller τ) than in the stochastic models. Changing the coalescent rate to be proportional to 1/(*N*(*t*) − 1) instead of 1/*N*(*t*) does not change the probability density (blue dotted line).

We performed additional numerical experiments to investigate whether using the expected population size conditioned on survival, *N*_BD_(*t*) (equation (2.2)), instead of the usual expression in equation (2.5) improves the approximation of the CD to the BD model (light blue line; figures 2 and 3; electronic supplementary material, figures S1–S5). As *N*_BD_(*t*) is not defined for *τ* > *T*, we plot only the distribution function in [0, *T*], with the value at *T* denoting the percentage of pairs having coalesced within [0, *T*]. As expected, for all considered parameter combinations, the modification of population size makes coalescent times older than under the CD model. However, for all considered parameter combinations, coalescent times are also older than under the BD model, so this new setting over-corrects the deterministic coalescent even for small *R*_0_ (when we expect barely any events at *τ* > *T*). Thus, it appears that ignoring full stochasticity in the coalescent population size introduces biases.

### (b) Stochastic coalescent

The cumulative probability distribution of the CS model is plotted as a red line. Again, the value at *T* denotes the percentage of pairs having coalesced within [0, *T*]. For small values of *R*_0_, the CS model is a good approximation to the BD model. As *R*_0_ increases, the CS line flattens out compared with the BD line close to time *T*. The amplified effect of flattening out for high *R*_0_ is explained by more coalescent events happening ancestral to *T* for increasing *R*_0_; this higher proportion for increasing *R*_0_ is quantified in the deterministic case above. Using a coalescent rate proportional to 1/(*N*(*t*) − 1) rather than to 1/*N*(*t*) results in more recent coalescent events. In particular, each pair has coalesced by time *T* with probability 1. However, for small *N*, coalescent events now happen more recently than under the BD model.

We point out that when doing phylogenetic inference of population dynamic parameters, we propose a time of origin *T* and a set of coalescent times, which are on interval [0, *T*]. Thus, the prior distributions on coalescent times are the coalescent time distributions plotted in figures 2 and 3 and electronic supplementary material, figures S1–S5, but normalized to be 1 at time *T*.

## 4. Discussion

We show here that deterministic coalescent approaches both under- and overestimate the coalescent times of two randomly sampled lineages depending on *R*_0_, with a stochastic BD model being used as a model of reference. Parameter estimates obtained using the coalescent approximation must therefore be treated with caution, because it is not immediately clear how strong the under- or overestimation is. The reason for the bias for small *R*_0_ is the stochastic change in population size, which is ignored by the deterministic coalescent. A stochastic version of the coalescent can correct the bias for small values of *R*_0_ and large enough population sizes (*N* > 100 for *R*_0_ = 1.05 and *N* > 1000 for *R*_0_ = 1.3 and *R*_0_ = 1.6). The reason for the remaining bias for larger *R*_0_ is that the coalescent approximation allows for the two lineages to coalesce after a time that is longer than the duration of the population growth process (i.e. the time at which the population size was 1).

More generally, any coalescent approximation (either parametric or non-parametric) that uses a population size function that decreases to a small number at any given time at which two or more lineages have not yet coalesced should be avoided. This has already been highlighted by Griffiths & Tavaré [14, p. 404]: ‘there are cases in which variable population size processes are better studied in their original, discrete timescale, particularly those which have very small population sizes for many generations. Although we do not explicitly examine such cases in this paper, the methods developed here can be exploited in that setting too.’ Griffiths & Tavaré nevertheless fitted the coalescent with a deterministic exponentially growing population to a dataset (which is fine in case the number of generations where the population size is ‘very small’ is considered to be not ‘many’) [14]. With the release of BEAST [38] assuming a coalescent-based prior on trees, the coalescent became the model of choice to use in phylodynamics, without validating its assumptions. We now show here that the exponential growth deterministic coalescent, however, may lead to biased growth rate estimates when studying exponentially growing populations in the presence of demographic stochasticity, questioning the accuracy of such growth rate estimates.

The appealing feature of the deterministic coalescent is that the likelihood of the population size *N*(*τ*) and generation time *ρ*(*τ*) (here constant, but see Volz *et al.* [8] for time-varying generation time) for a given tree is easily calculated by tracing all lineages backwards in time, with the lineages coalescing at rate 1/(*N*(*τ*)*ρ*(*τ*)). The computational gain, however, comes at the cost of incorrect estimates if the true population growth dynamics are stochastic in nature, which is the case for most biologically relevant applications. A stochastic coalescent can partly correct these errors, but because the population size 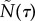 is an ensemble of realizations of a stochastic process, the likelihood must be computed by averaging across the whole ensemble. Thus, the likelihood computation must be repeated a large number of times, which negates any computational advantage of the coalescent approximation.

In fact, when the population size is specified by a single trajectory 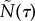, the computational cost of calculating the likelihood for a fixed tree under the BD model is of the same order as under the coalescent approximation. While the coalescent likelihood is calculated from the rate of coalescence 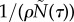, the BD likelihood is calculated in the following way: each birth and removal event in the tree must coincide with a birth and removal event in the trajectory, otherwise the likelihood is zero. An example for a tree with non-zero likelihood is the tree in figure 1*b*, given the trajectory 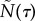 in figure 1*a*. If all birth events coincide, the probability of a tree given the trajectories is a product of simple combinatorial factors, one for each transmission and removal event in the trajectory. For a given event, the factor is either (i) the probability of the event occurring between individuals represented as lineages in the tree (if the event actually occurred on the tree) or (ii) the probability of the event occurring between other individuals outside of the tree (if the event actually occurred outside the tree).

In the particular case studied here—the constant-rate BD model—we can analytically integrate over all trajectories by using the closed-form solution for the coalescent time of two lineages provided in equation (2.4). This provides a computationally efficient way to infer population parameters using the fully stochastic model and avoiding any approximations.

The advantage of the deterministic coalescent is that the rates of coalescence of the sampled lineages backwards in time are easily derived from the population size function. As we described, this backward-in-time interpretation is equivalent when the exact population trajectory 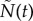 is known in the BD model and the CS model. It is unclear if such a simple backward-in-time (vertical) interpretation exists when averaging over trajectories. The BD model, however, has an alternative ‘horizontal’ interpretation of how sampled lineages coalesce when integrated over all trajectories, called the *point process representation* [27,39,40]. The point process allows us to sample the *n* − 1 coalescent times in a tree of *n* extant lineages from the same point process distribution independently. For tips labelled 1, 2, … , *n*, the *i*th draw from the point process distribution is the coalescent time of the tips *i* and *i* + 1. This horizontal interpretation allows one to quickly simulate a tree on *n* tips (namely by drawing *n* − 1 random variables, each from the same point process distribution). In inference, the likelihood of a tree on *n* tips is simply a product over the point process densities of each of the *n* − 1 coalescent times. Interestingly, the deterministic coalescent model does not have a horizontal point process representation [40]. It remains to be investigated if the stochastic coalescent has a horizontal point process representation.

We showed that coalescent models give rise to different coalescent time distributions for two lineages compared with BD models. This has important consequences both when simulating trees and when estimating population dynamic parameters from trees (such as the basic reproductive number *R*_0_ or the net growth of an epidemic *r* = *λ* − *μ*). Trees are simulated under the coalescent by starting at the present and fixing the present-day population size *N*_0_. Because the process time *T*_CD_ for the deterministic coalescent model is longer than the time *T*_BD_ for the BD model for small *R*_0_ (owing to the push of the past effect), the simulated trees will be older under the CD model than under the BD model with the same *N*_0_. As a consequence, when doing inference, if the true trees are realizations from a stochastic BD model with small *R*_0_, then growth rates estimated using the CD model will be overestimated, as the CD model expects older trees for the true growth rate. This is in agreement with previous results that, based on fixed trees, the deterministic coalescent overestimates growth rates and induces more ancestral coalescent events [9,17]. Furthermore, when doing inference, it has been observed that the deterministic coalescent is too confident in parameter estimates and underestimates the width of the highest posterior density (HPD) intervals [9,17]. As a consequence, the HPD intervals may not contain the true parameter with high probability when using the deterministic coalescent. This underestimate of parameter uncertainty is a drawback of the coalescent that is not apparent when considering the maximum-likelihood point estimates over a range of trees [41]. For more realistic epidemiological SIS- or SIR-type models, the pattern becomes more complex. Biases will again depend not only on *R*_0_, but also on the sampling scheme (i.e. sampled during exponential or post-exponential phase) and the overall population size [42].

Decisions have to be made about the sampling scheme in the BD model, and typically we assume that a fraction of individuals is sampled rather than two individuals from an arbitrary-sized population. Here, we conditioned on sampling two lineages from a population of *N*_0_ lineages, where *N*_0_ is a random variable and corresponds to the number of lineages in the population after time *T*. Different sampling schemes could be employed. A widely used sampling scheme is that each lineage in the present-day population is sampled with some probability *p*. The probability of sampling two individuals from a population of *N*_0_ at the present then is 
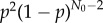
, which favours trajectories with few surviving lineages. The coalescent time distribution under the BD model will be affected by the specific choice of sampling. The distributions for the BD and CD models have been shown to be different in a number of cases [43].

The stochastic BD model considered here is only a crude approximation of real stochastic epidemic models. We showed that, even for this simple model, using a deterministic coalescent approximation to infer epidemiological parameters can lead to strong biases. We showed that a stochastic coalescent approximation yields correct parameter estimates for values of *R*_0_ close to 1. We do not expect the bias for large values of *R*_0_ to disappear when *N* → *∞,* because lineages coalesce with non-zero probability at times larger than the duration of the process. Thus, parameter estimates obtained using coalescent approximations should be treated with caution. Whenever possible, inference methods that assume a stochastic underlying population model should be used. Computationally tractable implementations of such models have only recently started to become available, such as for the SIS model [11], for skyline-type models [44] and for structured population models with stochastic exponential growth [45]. For more realistic epidemiological models, we must for now rely on coalescent-type approximations [8,10,19,21], or BD-based approximations, such as the BDSIR model [12]. This current dependence on approximations reveals the need for a stronger focus on developing exact methods, as well as thorough validation of approximate methods for epidemiological models.

## Supplementary Material

SI Text
