# Supplementary material for: How well can the exponential-growth coalescent approximate constant-rate birth–death population dynamics?
Source: Proc Biol Sci. 2015 May 7;282(1806):20150420. doi: 10.1098/rspb.2015.0420 (PMC4426635; doi:10.1098/rspb.2015.0420)
Supplement: SI Text [file rspb20150420supp1.pdf]

How well can the exponential-growth coalescent approximate constant rate birth-death population dynamics?
Supplementary Material

Tanja Stadler[1,2,*], Timothy G. Vaughan[3,4,5], Alex Gavruskin[3,†], Stephane Guindon[7,8,†], Denise Kühnert[1,2,6,†], Gabriel E. Leventhal[6,†], Alexei J. Drummond[3,4,*]

[1] Department of Biosystems Science and Engineering, ETH Zürich, Switzerland
[2] Swiss Institute of Bioinformatics (SIB), Switzerland
[3] Department of Computer Science, The University of Auckland, Auckland, New Zealand
[4] Allan Wilson Centre for Molecular Ecology and Evolution, New Zealand
[5] Massey University, Palmerston North, New Zealand
[6] Institute of Integrative Biology, ETH Zürich, Zürich, Switzerland
[7] Department of Statistics, The University of Auckland, Auckland, New Zealand
[8] LIRMM, CNRS Monpellier, France
[†] Alphabetical order
[*] Corresponding authors: tanja.stadler@bsse.ethz.ch & alexei@cs.auckland.ac.nz

Supplementary Methods

Derivation of $f_{BD}(\tau)$

In the following, we derive an analytical expression for $f_{BD}(\tau)$. We employ point process theory (Popovic, 2004; Gernhard, 2008). The population dynamic process of birth and death events until time T can be represented by an oriented tree (Ford et al., 2009). An oriented tree on n tips at time T, and all dead lineages being pruned, may be represented by a vector of $n-1$ coalescent events. Now, selecting two tips at random from the n tips and tracing back until their time of coalescence corresponds to choosing any block of successive entries from the vector of $n-1$ coalescent events uniformly at random, and the time of coalescence is the maximum of the coalescent events in the chosen block (Lambert and Stadler, 2013). The first order statistic of $i-1$ coalescent events is given by (Gernhard, 2008, Eq. 6) as

$$(i-1)F(\tau|T)^{i-2}f(\tau|T),$$

where $f(\tau|T) = \mu p_1(\tau)/p_0(T)$ and $F(\tau|T) = p_0(\tau)/p_0(T)$. There are $\binom{n}{2}$ possible block choices in a vector of $n-1$ entries. For block size $i-1$, there are $n-(i-1)$ possible ways to choose two lineages. Furthermore, the probability of obtaining n extant lineages after time τ is (Kendall, 1948),

$$p_n(\tau) = p_1(\tau)\left(\frac{\lambda}{\mu}p_0(\tau)\right)^{n-1}.$$

Conditioning on obtaining at least two lineages after time τ requires dividing $p_n(\tau)$ by $1-p_0(\tau)-p_1(\tau)$. Summing over all n and i yields

$$f_{BD}(\tau) = \sum_{n=2}^{\infty}\sum_{i=2}^{n}(i-1)F(\tau|T)^{i-2}f(\tau|T)\frac{n-(i-1)}{\binom{n}{2}}\frac{p_1(T)}{1-p_0(T)-p_1(T)}\left(\frac{\lambda}{\mu}p_0(T)\right)^{n-1}.$$

With $\xi = \frac{\lambda}{\mu}p_0(T) = \frac{\lambda-\lambda e^{-(\lambda-\mu)t}}{\lambda-\mu e^{-(\lambda-\mu)t}}$ and noting that $|\xi| < 1$ and $|\xi F(\tau|T)| = |\frac{\lambda}{\mu}p_0(\tau)| < 1$, we obtain for $f_{BD}(\tau)$,

$$
\begin{aligned}
f_{BD}(\tau) &= \sum_{n=2}^{\infty}\sum_{i=2}^{n}(i-1)F(\tau|T)^{i-2}f(\tau|T)\frac{n-(i-1)}{\binom{n}{2}}\frac{p_1(T)}{1-p_0(T)-p_1(T)}\left(\frac{\lambda}{\mu}p_0(T)\right)^{n-1} \\
&= \frac{f(\tau|T)p_1(T)}{F(\tau|T)(1-p_0(T)-p_1(T))}\sum_{n=2}^{\infty}\frac{1}{\binom{n}{2}}\left(\frac{\lambda}{\mu}p_0(T)\right)^{n-1}\sum_{i=1}^{n-1}iF(\tau|T)^{i}(n-i) \\
&= \frac{f(\tau|T)p_1(T)}{F(\tau|T)(1-p_0(T)-p_1(T))}\sum_{n=2}^{\infty}\frac{1}{\binom{n}{2}}\left(\frac{\lambda}{\mu}p_0(T)\right)^{n-1} \\
&\quad\times\Bigg(n\frac{(n-1)F(\tau|T)^{n+1}-nF(\tau|T)^{n}+F(\tau|T)}{(F(\tau|T)-1)^2} \\
&\qquad -\frac{1}{(F(\tau|T)-1)^3}\Big[(n-1)^2F(\tau|T)^{n+2}-(2n^2-2n-1)F(\tau|T)^{n+1} \\
&\qquad\quad +n^2F(\tau|T)^{n}-F(\tau|T)^2-F(\tau|T)\Big)\Big] \\
&= \frac{2f(\tau|T)p_1(T)}{(1-p_0(T)-p_1(T))(F(\tau|T)-1)^3} \\
&\quad\times\sum_{n=2}^{\infty}\Bigg\{\frac{1}{n(n-1)}\left(\frac{\lambda}{\mu}p_0(T)\right)^{n-1}\big(nF(\tau|T)^{n+1}-nF(\tau|T)^{n}+nF(\tau|T) \\
&\qquad -n-F(\tau|T)^{n+1}-F(\tau|T)^{n}+F(\tau|T)+1\big)\Bigg\} \\
&= \frac{2f(\tau|T)p_1(T)}{(1-p_0(T)-p_1(T))(F(\tau|T)-1)^3} \\
&\quad\times\Big[(F(\tau|T)+1)\frac{1-\xi}{\xi}(\ln(1-\xi)-1) \\
&\qquad -F(\tau|T)(F(\tau|T)+1)\frac{1-\xi F(\tau|T)}{\xi F(\tau|T)}(\ln(1-\xi F(\tau|T))-1) \\
&\qquad -(F(\tau|T)-1)\ln(1-\xi)-F(\tau|T)(F(\tau|T)-1)\ln(1-\xi F(\tau|T))\Big] \\
&= \frac{2f(\tau|T)p_1(T)}{(1-p_0(T)-p_1(T))(1-F(\tau|T))^3\xi} \\
&\quad\times\Big[(F(\tau|T)^2-1)\xi+F(\tau|T)(2\xi-1)(\ln(1-\xi)-\ln(1-\xi F(\tau|T))) \\
&\qquad +\ln(1-\xi F(\tau|T))-\ln(1-\xi)\Big] \\
&= \frac{2f(\tau|T)p_1(T)}{(1-p_0(T)-p_1(T))(1-F(\tau|T))^3} \\
&\quad\times\left(F(\tau|T)^2-1+\left(2F(\tau|T)-\frac{\mu F(\tau|T)+\mu}{\lambda p_0}\right)\ln\frac{\mu-\lambda p_0}{\mu-\lambda p_0 F(\tau|T)}\right) \\
&= \frac{2f(\tau|T)p_1(T)}{(1-p_0(T)-p_1(T))(1-F(\tau|T))^3} \\
&\quad\times\left(F(\tau|T)^2-1+\left(2F(\tau|T)-\frac{\mu}{\lambda}\frac{F(\tau|T)+1}{p_0(T)}\right)\ln\left(\frac{\mu-\lambda p_0(T)}{\mu-\lambda p_0(\tau)}\right)\right).
\end{aligned}
$$

We note that here we condition on sampling exactly two out of n tips. In previous work (Yang and Rannala, 1997; Stadler, 2010, 2013), it was assumed that each tip is sampled with a probability ρ. Thus the probability of sampling exactly two tips is $\binom{n}{2}\rho^2(1-\rho)^{n-2}$. The probability density of sampling two tips with coalescent time τ, now without conditioning on the process leading at least two extant lineages, is thus

$$f_{BD}(\tau|\rho) = (1 - p_0(T) - p_1(T)) \sum_{n=2}^{\infty}\sum_{i=2}^{n} \binom{n}{2}\rho^2(1-\rho)^{n-2}(i-1) \times F(\tau|T)^{i-2} f(\tau|T) \frac{n-(i-1)}{\binom{n}{2}} \frac{p_1(T)}{1-p_0(T)-p_1(T)} \left(\frac{\lambda}{\mu}p_0(T)\right)^{n-1}.$$

This expression for $f_{BD}(\tau|\rho)$ simplifies to Equation (1) in (Stadler, 2013).

Link between the *BD* and *CD* model

Under the coalescent model, when time is expressed in calendar units, the coalescent rate at time τ is $1/(N(\tau)\rho)$, with $N(\tau)$ being the population size at time τ. This means that the rate is defined not only by the population size $N(\tau)$, but also by a time-scale ρ, which, for a Wright-Fisher model, simply corresponds to the generation time.

On the other hand, under the birth-death process, the rate with which a single individual undergoes a birth event is λ. In a population with $N(\tau)$ individuals where each individual independently undergoes birth events at a rate λ, the total rate at which a birth event occurs is $\lambda N(\tau)$. The probability that a single forward-time birth event corresponds to the backward-time coalescence of two sampled lineages is $1/\binom{N(\tau)}{2}$. Thus the rate of coalescence of two lineages under the birth-death process is $\lambda N(\tau)/\binom{N(\tau)}{2} = 2\lambda/(N(\tau)-1)$. When $N(\tau)$ is large, $N(\tau)-1 \simeq N(\tau)$ and the rate of coalescence of the two lineages simplifies to $2\lambda/N(\tau)$.

We thus have two independent derivations of the coalescent rate at time τ, one under the coalescent and one under the birth-death model. For the coalescent to approximate the birth-death process, the following equality must hold,

$$\frac{1}{N(\tau)\rho} = \frac{2\lambda}{N(\tau)},$$

and hence $\rho = 1/(2\lambda)$. In the particular case where $\lambda = \mu$, ρ can be interpreted as the expected length of a branch in the genealogy of a population evolving under a birth-death process.

Derivation of $f_{CDN}(\tau)$

Given a population size $N_{BD}(t)$ from Equation (2) in main text, the modified coalescent rate measured in backward time, $\tau = T - t$, is

$$\frac{2\lambda}{N_{BD}(\tau)} = \frac{2\lambda r}{(\lambda - \mu e^{-rT} - \frac{\mu^2}{\lambda-\mu e^{-rT}}e^{-2rT})e^{rT}e^{-r\tau} + \frac{\mu(\lambda+\mu)}{\lambda-\mu e^{-rT}}e^{-rT} - \frac{\lambda\mu}{\lambda-\mu e^{-rT}}e^{-rT}e^{r\tau}}. \quad (1)$$

Thus, the coalescent time probability density under the coalescent with population size function $N_{BD}(\tau)$ is,

$$f_{CDN}(\tau) = \frac{2\lambda}{N_{BD}(\tau)} e^{-\int_0^{\tau} \frac{2\lambda}{N_{BD}(u)} du}.$$

To derive the explicit form of $f_{CDN}(\tau)$, let us denote the coefficients

$$\begin{aligned} c_0 &= (\lambda - \mu e^{-rT} - \frac{\mu^2}{\lambda - \mu e^{-rT}} e^{-2rT}) e^{rT}, \\ c_1 &= \frac{\mu(\lambda + \mu)}{\lambda - \mu e^{-rT}} e^{-rT}, \\ c_2 &= -\frac{\lambda\mu}{\lambda - \mu e^{-rT}} e^{-rT}. \end{aligned}$$

The coalescent rate $2\lambda/N_{BD}(\tau)$ from Equation (1) becomes $2\lambda r/(c_2 e^{r\tau} + c_1 + c_0 e^{-r\tau})$. Further, let

$$g(\tau) := \int_0^{\tau} \frac{2\lambda}{N_{BD}(\tau)} d\tau = 2\lambda \int_0^{\tau} \frac{r e^{r\tau} d\tau}{c_2 (e^{r\tau})^2 + c_1 e^{r\tau} + c_0} = 2\lambda \int_0^{\tau} \frac{1}{c_2 (e^{r\tau})^2 + c_1 e^{r\tau} + c_0} de^{r\tau}.$$

The result of integration is,

$$g(\tau) = \begin{cases} \frac{2\lambda}{\sqrt{c_1^2 - 4c_2c_0}} \ln \left| \frac{(2c_2 e^{r\tau} + c_1 - \sqrt{c_1^2 - 4c_2c_0})(2c_2 + c_1 + \sqrt{c_1^2 - 4c_2c_0})}{(2c_2 e^{r\tau} + c_1 + \sqrt{c_1^2 - 4c_2c_0})(2c_2 + c_1 - \sqrt{c_1^2 - 4c_2c_0})} \right| & \text{if } 4c_2c_0 - c_1^2 < 0, \\ \frac{4\lambda}{2c_2 + c_1} - \frac{4\lambda}{2c_2 e^{r\tau} + c_1} & \text{if } 4c_2c_0 - c_1^2 = 0, \\ \frac{4\lambda}{\sqrt{4c_2c_0 - c_1^2}} (\arctan \frac{2c_2 e^{r\tau} + c_1}{\sqrt{4c_2c_0 - c_1^2}} - \arctan \frac{2c_2 + c_1}{\sqrt{4c_2c_0 - c_1^2}}) & \text{if } 4c_2c_0 - c_1^2 > 0, \end{cases}$$

which establishes

$$f_{CDN}(\tau) = \frac{2\lambda}{N_{BD}(\tau)} e^{-g(\tau)}.$$

References

Daniel Ford, Frederick A Matsen, and Tanja Stadler. A method for investigating relative timing information on phylogenetic trees. *Syst Biol*, 58(2):167–83, Apr 2009. doi: 10.1093/sysbio/syp018.

Tanja Gernhard. The conditioned reconstructed process. *J Theor Biol*, 253(4):769–78, Aug 2008. doi: 10.1016/j.jtbi.2008.04.005.

David G. Kendall. On the generalized “birth-and-death” process. *Ann Math Stat*, 19(1): 1–15, Mar 1948.

Amaury Lambert and Tanja Stadler. Birth-death models and coalescent point processes: the shape and probability of reconstructed phylogenies. *Theor Popul Biol*, 90:113–28, Dec 2013. doi: 10.1016/j.tpb.2013.10.002.

Lea Popovic. Asymptotic genealogy of a critical branching process. *Ann Appl Probab*, 14 (4):2120–2148, 2004. doi: 10.1214/105051604000000486.

Tanja Stadler. Sampling-through-time in birth-death trees. *J Theor Biol*, 267(3):396–404, Dec 2010. doi: 10.1016/j.jtbi.2010.09.010.

Tanja Stadler. How can we improve accuracy of macroevolutionary rate estimates? *Syst Biol*, 62(2):321–329, 2013. doi: 10.1093/sysbio/sys073.

Ziheng Yang and Bruce Rannala. Bayesian phylogenetic inference using DNA sequences: a markov chain monte carlo method. *Mol Biol Evol*, 14(7):717–724, 1997.

Supplementary Figures

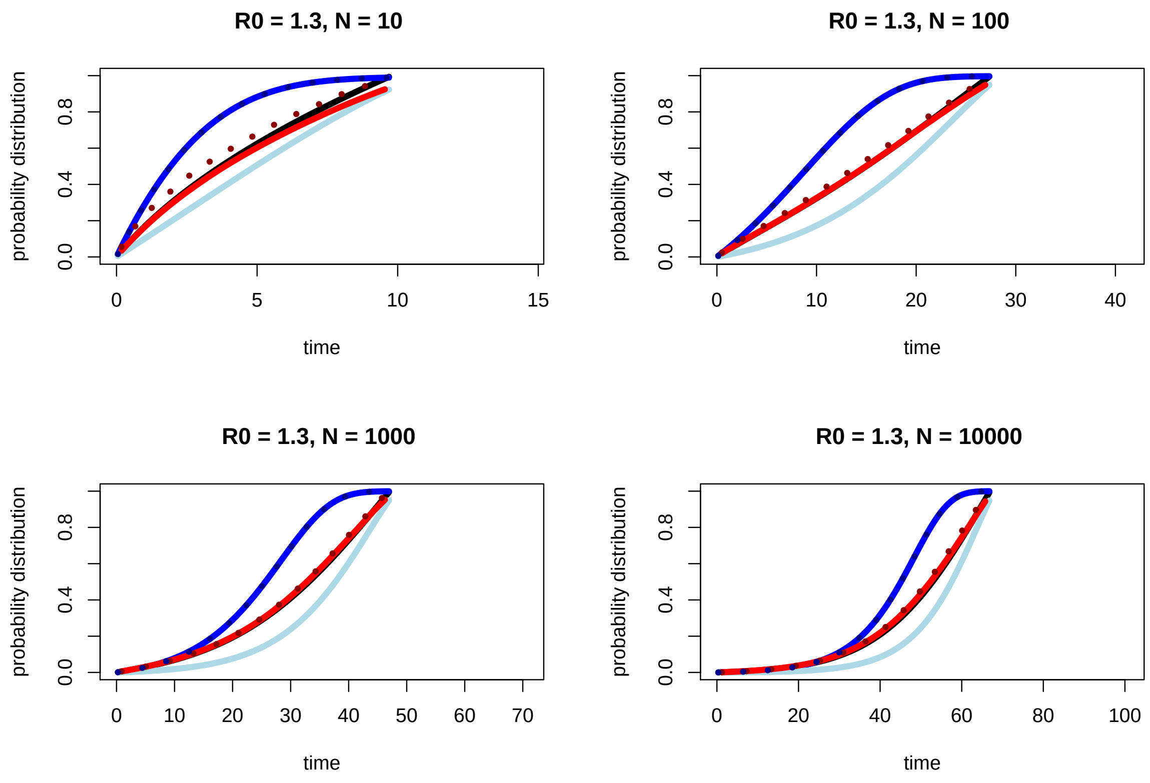

Supp. Fig. 1: Cumulative probability distribution function of time to coalescence for $R_0 = 1.3$ and $N = 10, 100, 1000, 10000$. For details see caption of Figure 2.

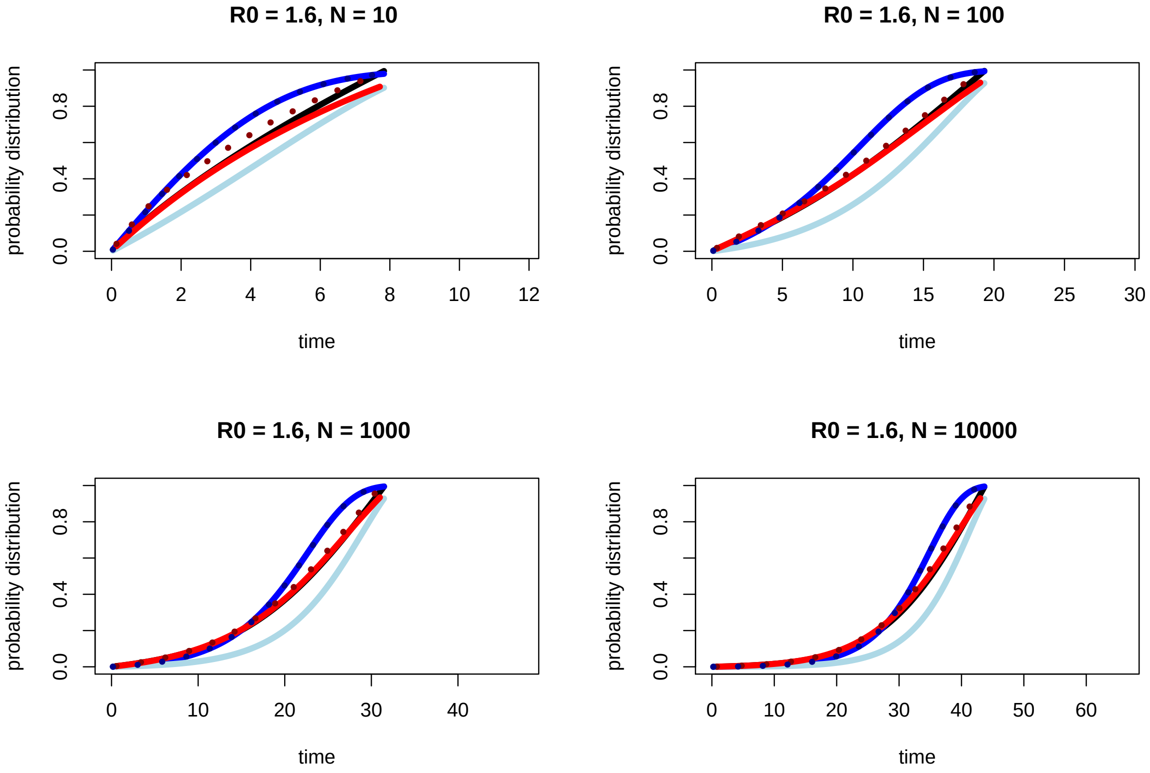

Supp. Fig. 2: Cumulative probability distribution function of time to coalescence for $R_0 = 1.6$ and $N = 10, 100, 1000, 10000$. For details see caption of Figure 2.

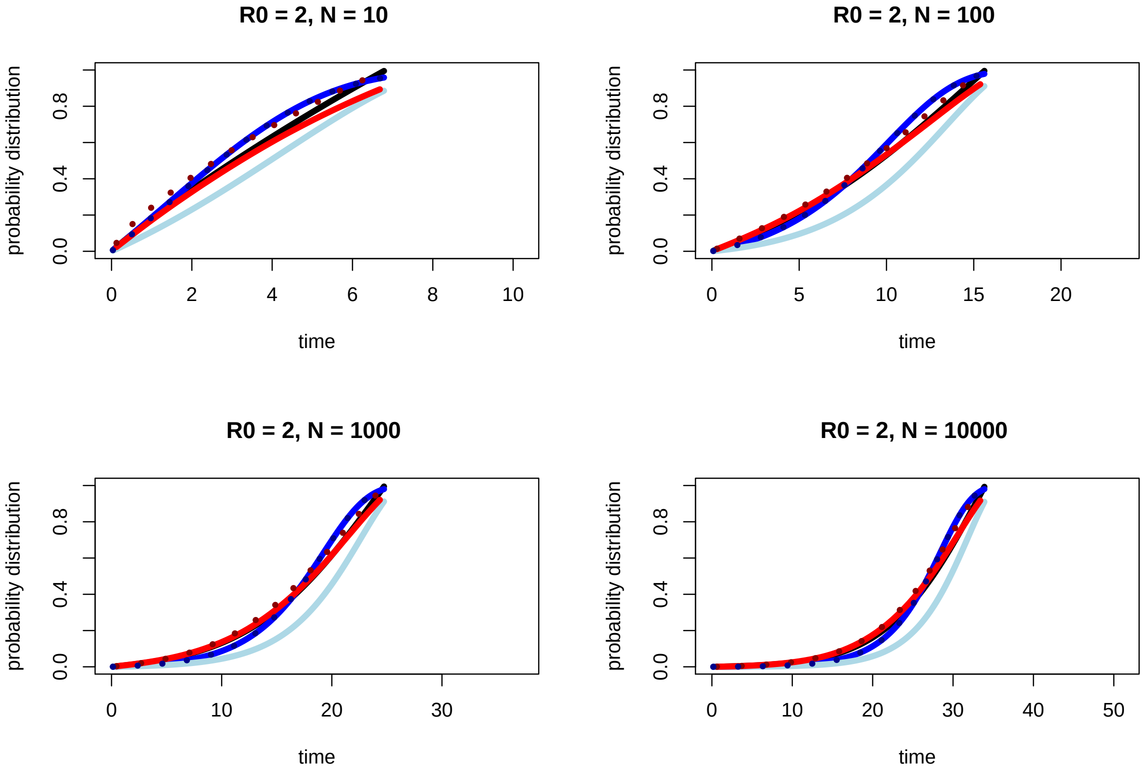

Supp. Fig. 3: Cumulative probability distribution function of time to coalescence for $R_0 = 2.0$ and $N = 10, 100, 1000, 10000$. For details see caption of Figure 2.

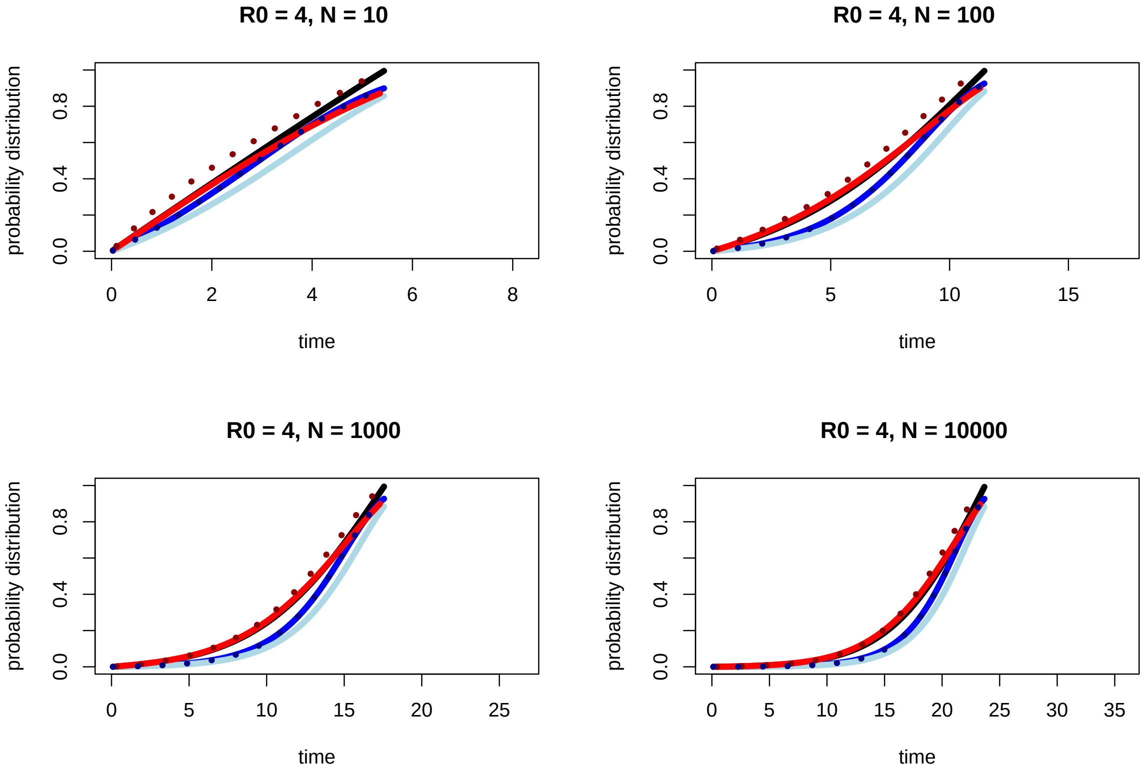

Supp. Fig. 4: Cumulative probability distribution function of time to coalescence for $R_0 = 1.3$ and $N = 10, 100, 1000, 10000$. For details see caption of Figure 2.

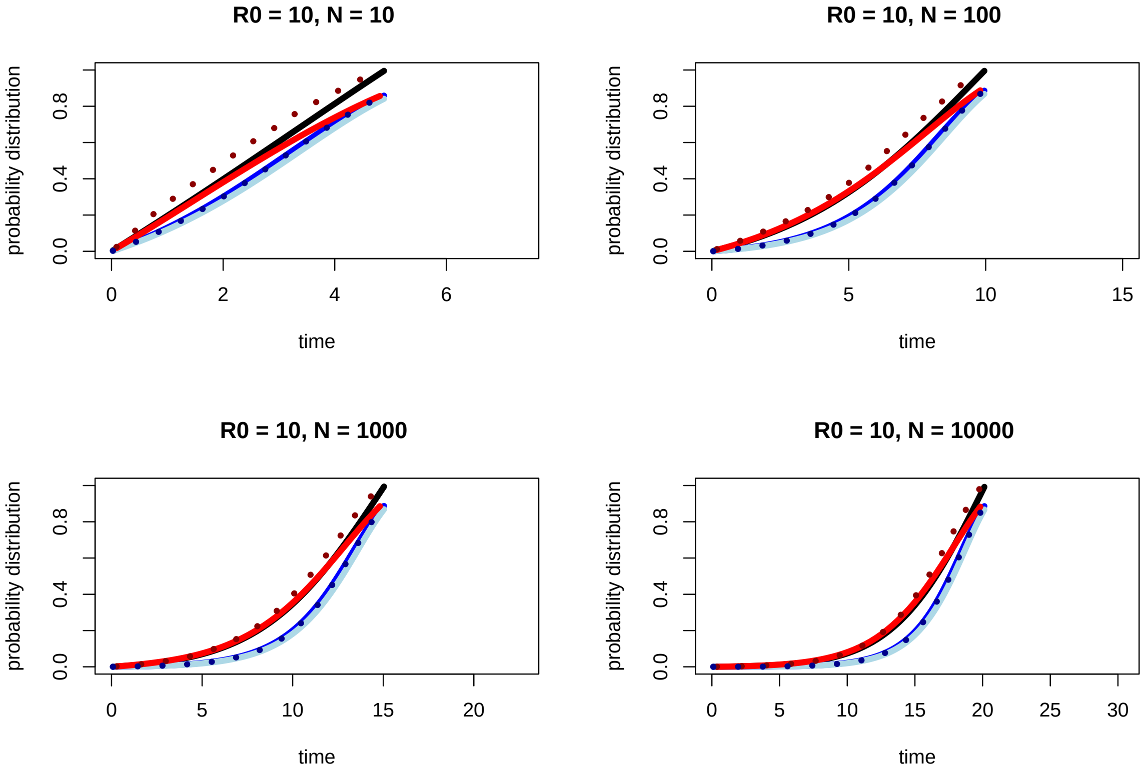

Supp. Fig. 5: Cumulative probability distribution function of time to coalescence for $R_0 = 10$ and $N = 10, 100, 1000, 10000$. For details see caption of Figure 2.
